# Electroacupuncture for postoperative pain management after total knee arthroplasty: Protocol for a systematic review and meta-analysis

**DOI:** 10.1097/MD.0000000000010014

**Published:** 2018-03-02

**Authors:** Jie Xiong, Huiying Li, Xiaoya Li, Lihe Wang, Pengfei Zhao, Dongfang Meng, Zong xing Wei, Taotao Tian

**Affiliations:** a The First Affiliated Hospital of Henan University of Traditional Chinese Medicine; b Armed Police Corps Hospital of Henan Province, Zhengzhou; c Luoyang Orthopedic Hospital of Henan Province, Luoyang, China.

**Keywords:** electroacupuncture, postoperative pain, protocol, systematic review, total knee arthroplasty

## Abstract

**Background:**

Total knee arthroplasty (TKA) is one of the most common joint replacement surgeries in the United States. Postoperative pain is still a major complication after TKA. Electroacupuncture (EA) has been commonly used in clinical practice for pain after TKA, but its effects and safety remain uncertain. This protocol is described for a systematic review to investigate the beneficial effects and safety of EA for postoperative pain after TKA.

**Methods:**

Randomized controlled trials (RCTs) related to EA treatment of pain after TKA will be collected from 3 databases of English literature, namely PubMed, Embase, and Cochrane Library, and 4 databases of Chinese literatures, namely CBM, CNKI, VIP and Wanfang database. The retrieved trials will be those published from the time when the respective databases were built to January 2018. The therapeutic effects according to the change from baseline in the amount of pain measured by the visual analogue scale (VAS) or numerical rating scale, will be accepted as the primary outcomes. We will use RevMan V.5.3 software as well to compute the data synthesis carefully when a meta-analysis is allowed.

**Results:**

This systematic review and meta-analysis will provide a high-quality synthesis of current evidence of EA for pain after TKA.

**Conclusion:**

The conclusion of our systematic review will provide evidence to judge whether EA is an effective intervention for patient with postoperative pain after TKA.

**PROSPERO registration number:**

PROSPERO CRD 42018082407.

## Abstract 2

Supplemental Digital Content is available in the text

## 1 Introduction

Total knee arthroplasty (TKA) is one of the most common joint replacement surgeries in the United States. More than 600,000 patients in the United States have underwent TKA by 2010.^[1]^ The annual increase rate of TKA cases is projected to be 673% by 2030 in the United States as the surgery becomes increasingly common.^[2]^ TKA can be used to treat all kinds of diseases that affect the knees to alleviate pain and restore their function and help patients return to activity. In the United States, >95% of TKA patients suffer from osteoarthritis.^[3]^

Postoperative pain is still a major complication after TKA.^[4]^ However, pain-relief drugs, especially opioid analgesics, are associated with complications including drowsiness, vomiting, nausea, and respiratory depression.^[5,6]^ Therefore, we need to explore a safe and effective treatment for postoperative pain.

Complementary and alternative medicine (CAM) is increasingly accepted by both the developing and developed world as a substitute for conventional therapies.^[7]^ A third of Americans seek CAM treatment every year due to the poor effect of conventional medicine.^[8]^ As an important part of CAM, traditional Chinese medicine (TCM) is based on the theory of Yin-Yang, which is to maintain health via keeping energy balanced in the body.^[9]^ Originating in ancient China, acupuncture has been an important TCM practice where needles are inserted into specific points on human body, including torso, limbs, scalp, and auricle. According to the theories of TCM, acupuncture can help correct imbalance of flow of energy and relieve symptoms by stimulating different acupuncture points.^[10]^ Acupuncture has been widely adopted to treat pain in Asian countries.^[11]^ The National Institutes of Health suggests that acupuncture can be used as a complementary or alternative treatment for many diseases, among which include postoperative pain.^[12]^ Clinical studies have shown that acupuncture can help alleviate postoperative pain, reduce the dosage of opioid analgesics, and other related side effects.^[13]^ However, the mechanism of acupuncture analgesia remains undefined.

Electroacupuncture (EA) is a type of acupuncture that uses a special device to transmit electric current to the appropriate needles.^[14]^ Previous studies demonstrated that using low doses of traditional painkillers with EA could effectively alleviate pain and reduce the adverse effects of the drugs, such as tiredness.^[15]^ Although there are many clinical reports on the EA treatment of pain after TKA,^[16,17]^ a systematic evaluation and meta-analysis of its efficacy is still lacking. Adopting the method of evidence-based medicine (EBM), this study aims to evaluate and analyze the clinical randomized controlled trials of EA treatment of pain after TKA published globally, and to provide evidence for the clinical effectiveness of EA treatment of pain after TKA.

## 2 Methods

### 2.1 Inclusion criteria for study selection

#### 2.1.1 Types of studies

All randomized controlled trials (RCTs) will be included in the research.

#### 2.1.2 Types of patients

Patients undergoing TKA will be included.

#### 2.1.3 Types of interventions

The treatment group will be treated with EA, with no limits on the material of needles, acupuncture points for treatment, practice, retaining time of needles, and treatment course. Meanwhile, the control group will be treated with conventional pain relief drugs such as Celebrex or false EA therapy.

#### 2.1.4 Types of outcome measures

##### 2.1.4.1 Primary outcomes

The primary outcome is the change from baseline in the amount of pain measured by the visual analogue scale (VAS) or numerical rating scale.

##### 2.1.4.2 Secondary outcomes

- Functional evaluation of knee joint.
- The range of motion of the knee joint.
- Frequency and nature of adverse events.

### 2.2 Search methods for the identification of studies

#### 2.2.1 Electronic searches

RCTs related to EA treatment of pain after TKA will be collected from 3 databases of English literature, namely PubMed, Embase, and Cochrane Library, and 4 databases of Chinese literatures, namely CBM, CNKI, VIP, and Wanfang database. The retrieved trials will be those published from the time when the respective databases were built to January 2018. The combination of keywords and free words will be used to conduct the data collection, and the retrieval strategy will be determined based on several pre-retrievals. The keywords of retrieval will be electroacupuncture, TKA, and randomized. To ensure a high recall ratio, the references of the included studies and systematic reviews related to the subject will be added as the supplementary literature. The search strategy of PubMed will be shown in Appendix A.

#### 2.2.2 Searching other resources

In the meantime, we will also search medical journals in university libraries related to this topic, such as Journal of Clinical Acupuncture and Moxibustion (1985–2018.1), Chinese Acupuncture, and Moxibustion (1981–2018.1).

### 2.3 Data collection and analysis

#### 2.3.1 Selection of studies

**Figure 1:**
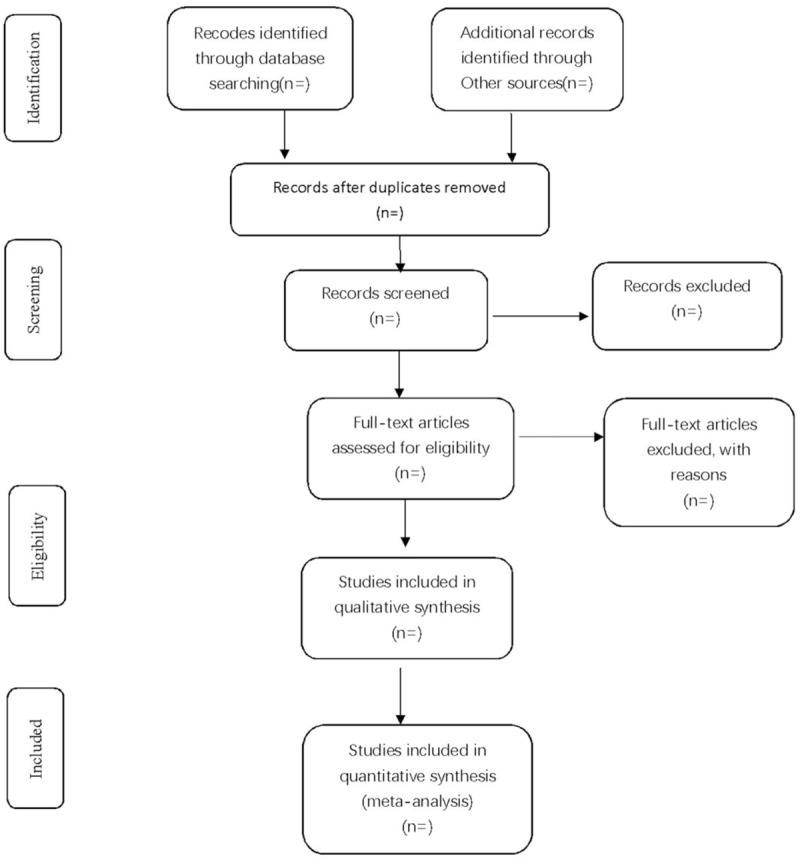
Flow diagram of study selection process.

Two researchers will independently remove the repeated data from the retrieved trails using the literature management system of EndnoteX7. They will then exclude the studies that are obviously not up to the standard by reading the titles and abstract, and decide the final inclusion of literature by reading the full text, group discussion, and contacting with the author to learn about the details of related researches. The final list of included references will be imported to a Microsoft Excel form. Both the literature search and the literature screening will be independently carried out by 2 research members. Another researcher will resolve the inconsistencies and check the last included literature should the need arise. The process of studies selection is presented in a PRISMA flow diagram (Fig. 1).

#### 2.3.2 Data extraction and management

Two researchers will extract the data using the software EpiData 3.1 for double entry. Another researcher will examine whether the extracted data are consistent and confirm the final retrieved data, including diagnosis, comorbidity, course, stage and severity of disease, sample size, age, sex intervention, specific therapy adopted in the control group, follow-up, outcome indicators, results of the study, and adverse events. The research members will discuss and contact the authors for missing data, errors and/or uncertainties, or settle the related disputes by third party arbitration.

#### 2.3.3 Assessment of risk of bias in included studies

The risk of bias in the included RCTs will be evaluated by 2 trained researchers independently from 7 dimensions, namely random sequence generation, allocation concealment, blinding method for patients, researchers and outcomes assessors, incomplete result data and selective reports, based on the bias risk assessment tools provided by Cochrane Handbook for Systematic Reviews of Interventions. The evaluation results will be divided into 3 categories: low risk, unclear, and high risk. In case of disagreement and inconsistency, the researchers will discuss in the group, contact the author to determine the details, and seek third party arbitration.

#### 2.3.4 Measures of treatment effect

The enumeration data will be indicated by the relative risk (RR) and the measurement data the mean difference (MD). Meanwhile, a 95% confidence interval (CI) will be used to show the effect sizes.

#### 2.3.5 Dealing with missing data

If there is unavailable data in an included trial, the researchers will attempt to obtain information by contacting the corresponding author. The analysis will be based on the available data if they fail to reach the author for the missing data.

#### 2.3.6 Assessment of heterogeneity

Chi-squared test (*α* = 0.1) and *I*^2^ value will be adopted respectively to analyze and determine the heterogeneity of the results of included researches. If *I*^2^ ≤50%, it can be deemed that the statistic heterogeneity among trials is negligible, and the fixed effects model will be employed to calculate the effect sizes. Otherwise the heterogeneity among the trials can be considered significant.

#### 2.3.7 Assessment of reporting bias

The visual symmetry on a funnel plot will be generated to determine whether a publication bias exists when there are at least 10 trials included. If the bias cannot be determined according to the generated image, the researchers will use the software STATA 11.0 to perform the quantitative analysis of the Egger test.

#### 2.3.8 Data synthesis

The software RevMan 5.3 (The Cochrane Collaboration, Oxford, England) will adopted to carry out the meta-analysis. The fixed effect model will be used for the meta-analysis if there is no statistical heterogeneity among the results. If a statistic heterogeneity is found, however, the source of the heterogeneity shall be further analyzed. The meta-analysis will then be conducted using the random effect model with the effect of the obvious clinical heterogeneity excluded. Where there is obvious clinical heterogeneity, the researchers can perform the subgroup or sensitivity analysis, or only descriptive analysis instead.

#### 2.3.9 Subgroup analysis

The researchers will conduct subgroup analysis according to patients’ age, sex, acupuncture points, and course of treatment if a relatively significant heterogeneity is observed in the included studies.

#### 2.3.10 Sensitivity analysis

The robustness of the results of the meta-analysis will be assessed based on whether the appropriate randomized and blinding methods are adopted to carry out the subgroup analysis.

#### 2.3.11 Dissemination and ethics

The protocol is not necessary for a formal ethical approval because the data are not individualized. It will be disseminated in a peer reviewed journal and presented at relevant conferences.

## 3 Discussion

Pain after TKA has significant negative effects on patients’ postoperative rehabilitation, daily activities, quality of life, social and economic conditions.^[16,17]^ EA is a potential effective and safe intervention to treat pain after TKA but lacks convincing and comprehensive evidence. Therefore, a high quality meta-analysis is needed for the comprehensive analysis of the existing clinical trials. The conclusions drawn from this systematic review will provide evidence of EBM for policy makers, patients, and clinicians to adopt the EA treatment of pain after TKA.

On the other hand, the study may have some limitations. Specifically, different forms of EA and quality of methodology adopted in the study can lead to significant heterogeneity. In addition, only English and Chinese medical databases will be searched for included RCTs since those are the only languages that the research members can understand. As a result, studies in other languages, such as Korean and Japanese, may be missed.

## Supplementary Material

Supplemental Digital Content
