# Supplementary material for: Electroacupuncture for postoperative pain management after total knee arthroplasty: Protocol for a systematic review and meta-analysis
Source: Medicine (Baltimore). 2018 Mar 2;97(9):e0014. doi: 10.1097/MD.0000000000010014 (PMC5851748; doi:10.1097/MD.0000000000010014)
Supplement: Supplemental Digital Content [file medi-97-e0014-s001.docx]

**Appendix A. Search strategy used in PubMed database**

#1 Arthroplasties, Replacement, Knee OR Arthroplasty, Knee Replacement OR Knee Replacement Arthroplasties OR Knee Replacement Arthroplasty OR Replacement Arthroplasties, Knee OR Knee Arthroplasty, Total OR Arthroplasty, Total Knee OR Total Knee Arthroplasty OR Replacement, Total Knee OR Total Knee Replacement OR Knee Replacement, Total OR Knee Arthroplasty OR Arthroplasty, Knee OR Arthroplasties, Knee Replacement OR Replacement Arthroplasty, Knee OR Arthroplasty, Replacement, Partial Knee OR Unicompartmental Knee Arthroplasty OR Arthroplasty, Unicompartmental Knee OR Knee Arthroplasty, Unicompartmental OR Unicondylar Knee Arthroplasty OR Arthroplasty, Unicondylar Knee OR Knee Arthroplasty, Unicondylar OR Partial Knee Arthroplasty OR Arthroplasty, Partial Knee OR Knee Arthroplasty, Partial OR Unicondylar Knee Replacement OR Knee Replacement, Unicondylar OR Partial Knee Replacement OR Knee Replacement, Partial OR Unicompartmental Knee Replacement OR Knee Replacement, Unicompartmental

#2 electroacupuncture OR electro-acupuncture

#3 Randomized controlled trial OR clinical study OR Clin-ical Trial OR Controlled study OR Controlled Trial OR Random*Control* study OR random* Control* Trial

#1 AND #2 AND #3
